# The Relationship of Clinical, Cognitive and Social Measures in Schizophrenia: A Preliminary Finding Combining Measures in Probands and Relatives

**DOI:** 10.3233/BEN-2011-0350

**Published:** 2012-01-20

**Authors:** David Huepe, Rodrigo Riveros, Facundo Manes, Blas Couto, Esteban Hurtado, Marcelo Cetkovich, Maria Escobar, Viviana Vergara, Teresa Parrao, Agustin Ibañez

**Affiliations:** ^1^Laboratory of Cognitive Neuroscience and SociocognitionUniversidad Diego PortalesSantiagoChile; ^2^National Scientific and Technical Research Council (CONICET)Buenos AiresArgentina; ^3^Laboratory of Experimental Psychology and NeuroscienceInstitute of Cognitive Neurology (INECO) and Institute of NeuroscienceFavaloro UniversityBuenos AiresArgentina; ^4^Pontificia Universidad Católica de ChileSantiagoChile

**Keywords:** Schizophrenia, multiplex family, social cognition, neuropsychology, first degree relatives

## Abstract

This study examines performance of schizophrenia patients, unaffected relatives and controls in social cognition, cognitive and psychiatric scales looking for possible markers of vulnerability in schizophrenia. Performance of schizophrenia patients from multiplex families, first-degree relatives, and matched controls was compared and, subsequently, discriminant analysis method was used for identifying the best predictors for group membership. By using Multigroup Discriminant Analyses on the three groups, the best predictors were PANSS, Premorbid Adjustment Scale, Faux Pas test, and a face/emotion categorizing task. This model obtained 82% correct global classification, suggesting that the combination of psychiatric scales and neuropsychological/social cognition tesks are the best approach for characterizing this disease. Although preliminary, our results suggest that social cognition tasks are robust markers of schizophrenia family impairments, and that combining clinical, social and neuropsychological measures is the best approach to asses patients and relatives vulnerability.

## 1. Introduction

Schizophrenia is a pervasive condition characterized by heterogeneous clinical, functional and cognitive manifestations [65]. Recent research points towards conceiving schizophrenia as a neurodevelopmental disorder, following the complex interaction of environmental and genetic factors [47]. Neurocognitive and social skills impairments are core features of the disease, since they are present from the first episode, remain stable during the course of the illness and persists even when the symptoms are remitted [23]. However, there is an ongoing debate regarding the degree of heritability of these deficits. The concept of endophenotype has re-emerged expecting to facilitate the study of genetic and environmental factors and their contribution to the deficits observed in schizophrenia. Endophenotype refers to hereditable and quantifiable characteristics associated to a disorder, but also can be found in persons who have not manifested the disease [7]. Although endophenotype might be multifactorial in origin, it is considered as an intermediate state between genes and development indicating a tendency to express a condi-

ISSN 0953-4180/12/$27.50 © 2012 – IOS Press and the authors. All rights reserved tion [2]. In the search of endophenotype candidates, increasingly more familial studies have been conducted assessing separately either clinical, social cognition or cognitive deficits [24].

Studies have shown that both patients and healthy relatives are affected in cognitive tasks [21,42,45]. In social cognition, relatives have also been found impaired, using tasks like emotion recognition and social inference ([19,49] for a review, see [20]). These findings have suggested that social cognitive impairments observed both in patients and relatives reflect underlying factors associated with genetic vulnerability for the disease or shared environmental influences [26].

Family loading for psychopathology has also been studied in schizophrenia patients using psychiatric rating scales. Ritsner et al. [48] studied the emotional reactivity associated to genetic or familial liability to develop schizophrenia in multiplex families. This finding suggests that positive family history is associated with higher levels of emotional distress, and it is thought to point to generalized vulnerability [15,43].

However, despite the evidence for impaired performance in patients, a few studies have combined social cognition and cognitive measures and their relationship to psychiatric rating scales in patients and their unaffected relatives [26]. Schizophrenia phenotype, as a complex disorder, might be better described when behavioural, social cognition and cognitive measures are combined. Combining several tasks that can accurately discriminate between different cognitive domains is recommended in schizophrenia research [27]. Incidentally, searching for composite measures is the recommended approach to study the heterogeneity of the disease [50].

The first aim of this study was to detect underlying cognitive, social cognition, and clinical impairments in schizophrenic patients from multiplex families, first degree healthy relatives and controls. Secondly, we intended to combine these measures in order to look up for the best predictors of group membership (that is, patient, relative and control group), which might help to suggest genetic vulnerability using a discriminant analysis statistical method. We discriminated between these groups based on participant performance using neuropsychological, social cognition and clinical rating scores as predicting variables

## 2. Method

### 2.1. Participants

Fifteen clinically-stable medicated schizophrenic patients from multiplex families, fourteen healthy first-degree relatives, and nineteen controls took part in this study. The three groups were similar for age, level of education, and sex distribution. A summary of sample characteristics is presented in Table 1. Initial inclusion criteria for patient group were: (1) the diagnosis of paranoid schizophrenia according to DSM-IV-TR criteria, using Structured Clinical Interview for DSM-IV (SCID) [16] and confirmed with Schedules of Clinical Assessment in Neuropsychiatry (SCAN) [66] applied by a trained physician; and (2) the presence of one or more relatives with the diagnosis of schizophrenia (no greater than a third-degree relative), evidenced by the Family Interview for Genetic Studies (FIGS) applied to the relative [39]. All of the patients were receiving maintenance antipsychotic medication: 13 patients were receiving atypical antipsychotics, one was receiving typical antipsychotics, and one was receiving both types of antipsychotics. Healthy relatives had to be first-degree relatives. SCAN was applied to relatives for ruling out any psychiatric condition in this group. Age of all participants was between 20 and 55 years old in order to participate. All subjects gave written informed consent, and payment was given in exchange for participation.

Exclusion criteria were: other diagnosis in axis I except from generalized anxiety disorder, antecedent history of substance abuse, history of mental retardation, neurological disease, or any clinical condition that could affect cognitive performance. Patients were recruited at Schizophrenic units. Relatives were recruited through patient participation. Healthy controls were randomly recruited from a larger pool of volunteers who had neither a history of abuse of recreational drugs nor a family history of neurodegenerative or psychiatric disorders. The study was approved by the Hospital Ethics Committee. Fifteen patients were contributed from 12 multiplex families, 1 family contributed with three patients and 1 family contributed with two patients, the rest of the 10 families contributed with one patient each one. Of the 14 unaffected relatives, there were 6 siblings and 6 parents. 1 son and 1 daughter where included in the study (28 and 25 years old, respectively). The rationale for including them is that they were the only relatives available for these patients. In the patient group, 3 patients informed to be occasional cannabis users.

### 2.2. Measures

#### 2.2.1. Assessment of cognitive functioning

For cognitive assessment, general intellectual capabilities were evaluated by Raven's Coloured Progres-

Table 1
Summary of sample characteristic

| | Schizophrenia patients | Relatives | Controls | Fisher Exact test, F Anova value or Chi-square value (d.f.) | P value |
|---|---|---|---|---|---|
| n (male/female) | 15(12/3) | 14 (6/8) | 18(11/7) | | 0,12 |
| Age (Mean ± SD) | 37.57(12,16) | 45.79(12.47) | 40.5(12.82) | $F_{(2,45)} = 1.41$ | 0.25 |
| Level of Education | | | | $X^2(6) = 8.50$ | 0.17 |
| Primary Education: | 1 | 0 | 1 | | |
| Secondary Education: | 6 | 4 | 2 | | |
| Tertiary Education Uncompleted: | 6 | 4 | 10 | | |
| Tertiary Education Completed | 2 | 6 | 6 | | |
| Age of Onset (Mean ± SD) | 26.46(9.92) | | | | |
| Illness Duration (Mean ± SD) | 11.73(9.18) | | | | |
| Antipsychotic Medication (N) | | | | | |
| Typical | 1 | | | | |
| Atypical | 14 | | | | |
| Both | 1 | | | | |

sive Matrices [46]. TMT part A was given as a measure of speed processing, while TMT part B measured sequencing and mental flexibility [6].

### 2.2.2. *Social cognition measures*

As social cognition measures, Reading the Mind in the Eyes was used to assess the ability to recognize social emotions [4]. Emotional Morphing (EM) measured dynamic recognition of six basic emotions: happiness, disgust, sadness, surprise, fear and anger [62]. The software Morpher 3.0 was used to create a sequence of 20 intermediate states of facial expression for this test.

The ability to correctly identify a faux pas situation was measured by the Faux pas test [3,59]. Ten stories contained a faux pas and the other ten stories did not contain a faux pas (Control stories). In the stimuli stories containing a faux pas, the character committing the faux pas was unaware that they had said something inappropriate, while the person in the story hearing it might have felt hurt or insulted. The stories were placed in front of the participants, therefore reducing the demands on working memory When a faux pas was correctly identified subjects were also asked two additional questions to measure intentionality – that is, recognizing that the person committing the faux pas was unaware that they had said something inappropriate– and emotional attribution, in which participants should recognize that the person hearing the faux pas might have felt hurt or insulted.

To measure automatic emotional associations, the Emotional Implicit Association Task (Emotional IAT) was used. IAT [20] is an experimental method that measures the strength of associations. The Emotional IAT is a modified version of the IAT, in this simultaneous stimulus categorization task the subject is asked to classify the emotional valence of positive/negative words and the emotional valence of positive/negative faces. The task involved the presentation of happy ($n = 20$) and angry ($n = 20$) faces along with pleasant words ($n = 71$) and unpleasant words ($n = 72$). Stimuli validation can be found in Hurtado et al. [25]. The test-blocks comprised 100 stimuli. Faces were displayed for 100 ms and words for 300 ms. Some blocks had the same valence dimensions: negative (anger/unpleasant labels on one side of the screen) and positive (happiness/pleasant categories on the other). These were called compatible blocks. Blocks having the opposite association were called incompatible blocks. In this way, the incompatible blocks imply either an associative contextual effect of semantic interference with facial evaluation or an associative contextual effect of facial interference with semantic evaluation. The dependent variable were IAT score, which is a measure of interference effect, accuracy for categorizing compatible face/words, and accuracy for categorizing incompatible face/words. A more detailed description of cognitive, social cognition test, as well as psychiatric rating scales can be found in supplementary data.

### 2.2.3. *Psychiatric rating scales*

To assess clinical symptomathology, all participants completed a battery of psychiatry rating scales. Positive and Negative Symptoms Scale PANSS [28] was used to assess the presence the positive and negative symptoms, as well as general psychopathology. Structured Interview for Assesing Perceptual Anomalies (SIAPA) was given for evaluating senso- perceptual anomalies [8], since self-experienced perceptual anomalies have been posited as a fundamental disorder in schizophrenia and these alterations have been reported in multiplex schizophrenic families [36]. Depressive episodes in schizophrenia were appraised by Calgary Depression Scale for Schizophrenia (CDSS) [1]. Premorbid Adjustment Scale (PAS) assessed the degree of achievement of developmental goals throughout infancy, adolescence and adulthood [9]. This test was included in the battery, since premorbid functioning has been associated to familial liability in schizophrenia [64].

#### *2.2.4. Statistical analysis*

For statistical analysis, groups were created for patients, relatives, and controls. Group differences were analyzed by entering data into a one way analysis of variance (ANOVA). Analyses were carried out using R Project for Statistical Computing software version 2.11.1 (www.rproject.org). Tukey and Bonferroni post-hoc analyses were conducted for significant differences where appropriate.

Multigroup Discriminant function Analysis (MDA) with step inclusions method (Wilk's Lambda) was performed to compare three groups on cognitive, social cognition and psychopathological variables. For every significant discriminant function, we tested the level of prediction of the variables in every group studied across the module of classification of MDA's cases (according to size of the groups), using Statistical Package for Social Sciences (SPSS), version 17.0. MDA combines independent variables for selecting and assessing the discriminant power of combined measures (predictor variables) applied in each group. Once best predictors were selected, a final model was run without the selection method. MDA is based on factor analytic method, which can classify the participants in the groups according to the discriminative ability of selected predictors, and the results can be used to visually represent the position of groups relative to each other in a discriminant space. MDA is a one-way analysis technique used for studying multiple groups, not requiring posing equal sample size.

Discriminant analysis technique was chosen since it is used for classifying subjects into groups on the basis of a battery of measurements, as well as, on its parsimonious interpretation [58]. Among other statistical tests, discriminant analysis is a mathematical maximization procedure which makes it a nice choice for distinguishing between groups, especially when the dependent variable is not metric [22]. There is evidence that statistic models of classification in schizophrenia and also in other patient studies, are feasible to be performed with several variables in similar sample size studies [33,34,54,55,60]. Our data is consistent with those previous reports by showing an adequate classification even in a small sample size. Moreover, regarding the discriminant analysis, Meredith [38], Porebski [44], and Darlington et al. [12] have argued in favor of using the discriminant function-variable based on its greater stability of the correlations in small- or medium-sized samples. Hair et al. [22] have stated that, at least, five observations for each independent variable are needed, which is adequate for our design. This implies that our sample size provides have enough power for statistical analysis.

## 3. Results

### *3.1. Detection of cognitive, social cognition and clinical impairment in patients and relatives*

Mean scores of the three groups on neuropsychological tests, social cognition tasks and psychopathological scales are shown in Table 2. A more detailed description of these data is presented in results section in supplementary data. For neuropsychological, social cognition tests and psychiatric measures, scores were analyzed by entering the scores into a One-way ANOVA comparing performance of patients, relatives, and controls. No group differences were found ($F(2,45) = 1.51$; $p = 0.32$), indicating similar intellectual functioning across the three groups. In the TMT-A, no difference between the three groups were observed evidencing similar speed processing performance in this task ($F(2,45) = 2.26$; $p = 0.21$). However, in the TMT-B significant difference was found across the groups ($F(2,45)4.33$; $p < 0.05$). Tukey adjustments for multiple comparisons showed patients group to be impaired compared to controls. For social cognition tasks, Faux Pas test scores showed a significant difference between the three groups ($F(2,45) = 9.26$; $p < 0.001$). Tukey adjustments for multiple comparisons showed significant differences benefiting controls compared to patients ($p < 0.001$). Significant differences also existed between relatives and controls indicating a poorer performance of relatives ($p < 0.05$). No differences between patients and relatives were found ($p = 0.33$) indicating similar performance in both groups.

In stories containing a minor conflict, but not a faux pas, no difference was observed across the groups evidencing adequate comprehension of the task in the three groups ($F(2,45) = 0.56$; $p = 0.61$).

In an attempt of measuring comprehension of a Faux pas situation, a discrepancy score was calculated, rep-

Table 2
Performance in Raven Progressive Matrices, Reading the Eyes in the Mind test and Faux Pas test for schizophrenia patients, first-degree relatives and controls, showing mean scores (standard deviations are presented in brackets). Tukey and Bonferoni post –hoc analysis were carried out where appropriate

| | Schizophrenia patients ($n = 15$) | Relatives ($n = 14$) | Controls ($n = 18$) | ANOVA F value (d.f.) and significant post-hoc comparisons |
|---|---|---|---|---|
| Raven's Coloured Progressive Matrices | 29.6 (4.03) | 29.21 (6.9) | 32 (3.09) | $F_{(2,45)} = 1.51$ |
| Trail Making Test- A | 55.0 (24.8) | 41.5 (20.06) | 43.57 (21.9) | $F_{(2,45)} = 2.26$ |
| Trail Making Test- B | 141.86 (88.4) | 94.7 (55.1) | 85.0 (26.9) | $F_{(2,45)} = 4.33^{*}$ |
| | | | | Patients – Controls* |
| Reading the Mind in the Eyes Test | 24.53 (6.1) | 26.36 (7.2) | 27 (4.1) | $F_{(2,45)} = 0.77$ |
| Faux Pas Test | | | | |
| Faux pas score | 24.8 (14.9) | 30,07 (10.4) | 41.72 (9.6) | $F_{(2,45)} = 9.26^{***}$ |
| | | | | Relatives – Controls* |
| | | | | Patients – Controls*** |
| Composite Score | 0.62 (0.1) | 0.69 (0.08) | 0.81 (0.1) | $F_{(2,45)} = 13.10^{***}$ |
| | | | | Relatives – Controls** |
| | | | | Patients – Controls*** |
| Control related stories correctly identified | 8.8 (1.8) | 9.29 (1.6) | 9.28 (0.7) | $F_{(2,45)} = 0.56$ |
| Discrepancies Scores | 1.67 (1.5) | 1.43 (1.3) | 0.83 (0.8) | $F_{(2,45)} = 2.00$ |
| Emotional Morphing | | | | Emotion |
| | | | | $F_{(5,45)} = 32.5^{***}$ |
| Happiness | 7.2 (1.3) | 7.07 (1.8) | 7.8 (0.5) | |
| Disgust | 3.1 (0.8) | 3.7 (1.9) | 4.0 (1.9) | |
| Sadness | 4.2 (1.9) | 5.0 (1.9) | 4.7 (2.9) | |
| Surprise | 5.2 (2.5) | 6.2 (1.4) | 6.7 (1.3) | |
| Fear | 3.8 (2.5) | 3.5 (1.8) | 4.3 (2.2) | |
| Anger | 4.6 (1.9) | 5.5 (2.1) | 5.5 (1.5) | |
| Emotional IAT Score | −0.74 (0.3) | −0.99 (0.6) | −1.05 (0.6) | $F_{(2,45)} = 0.69$ |
| Compatible Face Accuracy | 0.75 (1.6) | 0.85 (1.8) | 0.91 (7.8) | $F_{(2,45)} = 3.54^{*}$ |
| | | | | Patient – Controls* |
| Compatible Word Accuracy | 0.85 (9.2) | 0.86 (1.1) | 0.93 (3.9) | $F_{(2,45)} = 4.38^{*}$ |
| | | | | Patient – Controls* |
| Incompatible Face Accuracy | 0.64 (2.4) | 0.67 (1.4) | 0.69 (2.2) | $F_{(2,45)} = 0.24$ |
| Incompatible Word Accuracy | 0.65 (1.6) | 0.67 (1.4) | 0.67 (2.3) | $F_{(2,45)} = 0.04$ |
| PANSS | | | | |
| Positive | 17.73 (6.06) | 7.57 (0.8) | 7.0 (0.0) | $F_{(2,45)} = 56.86^{***}$ |
| | | | | Patients – Controls*** |
| | | | | Patients – Relatives*** |
| Negative | 18,67 (6.2) | 7.57 (1.3) | 7.0 (0.0) | $F_{(2,45)} = 59.37^{***}$ |
| | | | | Patients – Controls*** |
| | | | | Patients – Relatives*** |
| General | 28.6 (10.4) | 18.64 (2.7) | 17.52 (1.3) | $F_{(2,45)} = 13.60^{***}$ |
| | | | | Patients – Controls*** |
| | | | | Patients – Relatives*** |
| SIAPA | | | | Modality |
| | | | | $F_{(4,45)} = 1.27$ |
| | | | | Group |
| | | | | $F_{(2,45)} = 7.97^{**}$ |
| | | | | Patients – Controls** |
| | | | | Patients – Relatives* |
| | | | | SIAPA x Group |
| | | | | $F_{(8,45)} = 1.35$ |
| Visual | 4.86 (1.9) | 3.21 (0.4) | 3.63 (2.1) | |
| Auditive | 4.73 (1.7) | 3.42 (0.9) | 3.1 (0.3) | |
| Olfactive | 4.46 (2.2) | 4.14 (1.6) | 3.05 (0.2) | |
| Gustative | 4,2 (2.1) | 3.42 (0.8) | 3.05 (0.2) | |
| Tactile | 4 (1.4) | 3.35 (1.1) | 3.15 (0.5) | |
| Calgary Depression Scale | 3.39 (2.8) | 1.64 (1.2) | 1.36 (1.3) | $F_{(2,45)} = 8.06^{**}$ |
| | | | | Patients – Controls** |
| | | | | Patients – Relatives** |
| Pre-morbid Adjustment Scale | 49.26 (17.8) | 25.07 (8.2) | 13.21 (6.6) | $F_{(2,45)} = 39.10^{***}$ |
| | | | | Relatives – Controls* |
| | | | | Patients – Controls*** |
| | | | | Patients – Relatives*** |

Mean (± SD); $^{*}P < 0.05$; $^{**}P < 0.01$; $^{***}P < 0.001$.

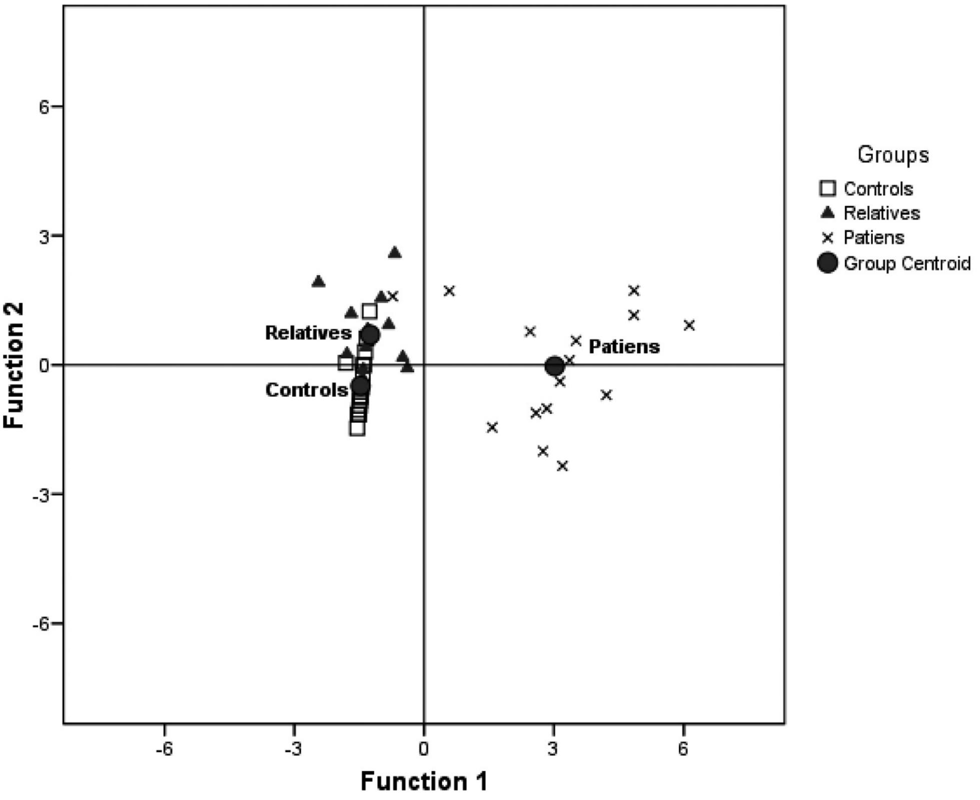

Fig. 1. Positions of groups centroids in function of two discriminant functions that maximizes individual separation. Functions 1 and 2 were derived from psychopathological predictors (SIAPA gustative scale, PANSS positive scale, PANSS negative scale and PAS scores). Group centroids illustrate clinical differences between probands and unaffected relatives.

resenting the number of discrepancies between the correct identification of the person saying something awkward and the belief that the speaker did know that the statement might be hurtful to the listener. The number of discrepancies revealed no differences in the ability to understand a faux pas situation for intergroup evaluation (F(2,45) = 2.009; $p = 0.18$).

Regarding global adjustment, significant difference was observed in PAS scores (F(2,45) = 39.106; $p < 0.001$) for intergroup evaluation. Post hoc comparisons evidenced a poorer adjustment of patients compared to relatives and controls (both, $p < 0.001$), but also lower level of achievement in relatives when they are compared to controls ($p > 0.05$).

### *3.2. Prediction of groups based on cognitive, social cognition and psychopathological performance*

#### *3.2.1. Prediction of group membership based on psychiatric rating scales data*

Main psychopathological measures were introduced into the analysis as independent variables, including SIAPA (visual, auditory, gustative, olfactive, and tactile scores), PANSS sub-scales, CDSS, and PAS scores. Group membership, that is patient, relative and control, was defined as dependent variable. Two discriminant functions were calculated between the predictors with a Wilks's $\lambda = 0.147$, $\chi^2$ (8) = 83.41, $p < 0.001$ for function 1, and Wilks's $\lambda = 0.799$, $\chi^2$ (3) = 9.79, $p < 0.05$ for function 2. The selected predictors are presented in Supplementary Table 1, Supplementary Data section, for each function with his respective standardized coefficients

This model obtained a correct global classification of 79.2% (similar percentage with cross-validation) accurately grouping 89.5% of the control group (17 out 19), 86.7% of the patients (13 out 15) and 57.1% of the relatives (8 out 14). In the relative group, the remaining 42.9% was incorrectly classified as a control. Although the classification percentage for relatives was lower, still it was largely superior to chance level (29%). Figure 1 illustrates how the discriminant functions and their respective centroids predict distinctions and similarities amongst the groups.

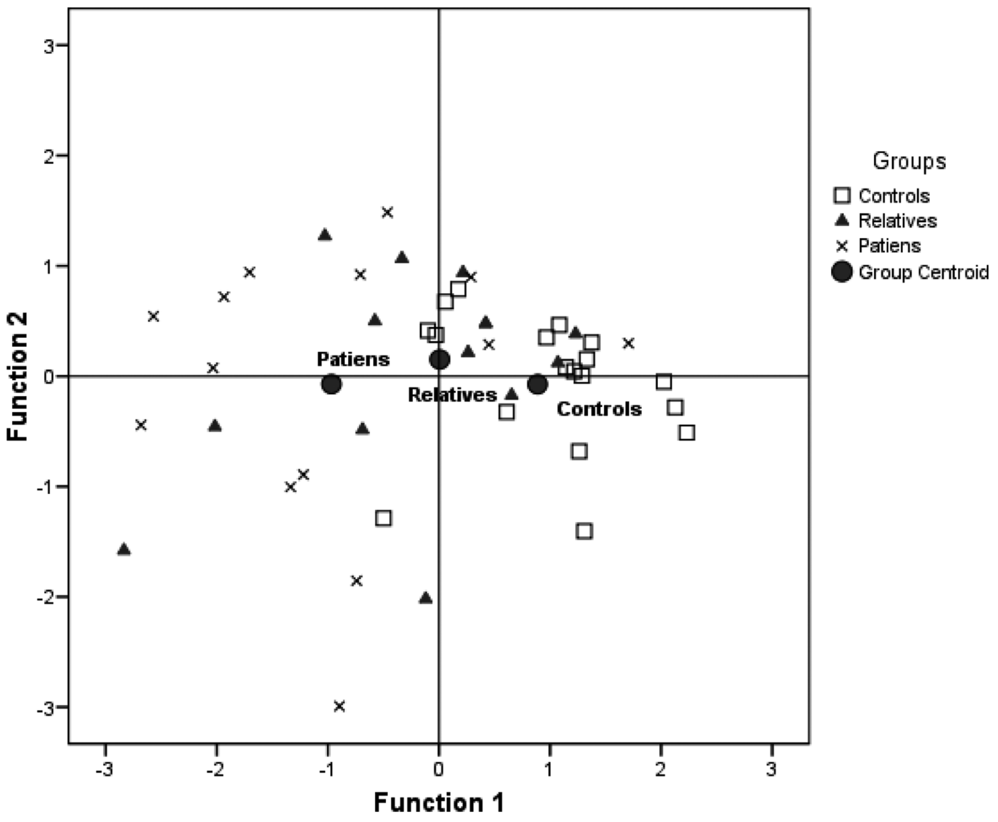

Fig. 2. Positions of group centroids in function of two discriminant functions that maximizes individual separation. Functions 1 and 2 were derived from cognitive and social cognition measures (Faux Pas, and IAT face congruent accuracy scores). Group centroids suggest similar performance in probands and relatives with respect cognitive, social cognition measures.

### *3.2.2. Prediction of group membership based on neuropsychological and social cognition tests data*

The set of predictors included cognitive and social cognition measures. The followings indexes were introduced in the analysis: TMT-A, TMT-B, Raven, Faux Pas and Reading the Mind scores, Emotional IAT dependent variables and EM scores for every emotion. Two discriminant functions were calculated with these predictors, with a Wilks's $\lambda = 0.605$, $\chi^2$ (4) = 16.81, $p < 0.01$ for function 1, and Wilks's $\lambda = 0.988$, $\chi^2$ (1) = 0.40, n.s. for function 2. Only two predictors were selected (presented in the Supplementary Table 2, Supplementary Data, with their respective standardized coefficients)

This model achieved a 63% correct global classification (58.7% using a cross validation). Of the control group, 72.2% was accurately classified (13 out 18), 71.4% of the patients (9 out 14) and only 42.9% of the relatives (6 out 14) were accurately grouped. In the relative group, 21.4% was wrongly classified as a control and 35.7% as patient. Although the accurate classification of relatives was lower in comparisons to the other groups, it was again greater than chance level (32%). This misclassification percentage of the relative group highlighted the similarities of patients and relatives regarding cognitive and social cognition impairments. Figure 2 illustrates the discriminant functions with their respective centroids.

### *3.2.3. Prediction of group membership combining neuropsychological, social cognition and psychopathological data*

A model combining the previously selected predictors was performed. It included SIAPA gustative scale, PANSS positive and negative scale, Faux pas scores and IAT Accuracy Face Compatible. All the predictors were entered without a selection method. Two discriminant functions were obtained with these predictors with a Wilks's $\lambda = 0.131$, $\chi^2$ (12) = 82.32; $p < 0.001$ for function 1, and Wilks's $\lambda = 0.686$, $\chi^2$ (5) = 15.29, $p < 0.01$ for function 2. In the Supplementary Table 3, Supplementary Data section, the structure matrix of the predictors of the model is presented

The factorial structure of the function 1 is associated to variables strongly related to clinical symptomatology. In other hand, the structure of the discriminant function 2 comprises cognitive and social cognition variables, including a psychopathological measure which correlates in both functions, although with a positive loading in the first function and with a negative loading in the second function.

This model showed 82% correct global classification (73% cross-validated), grouping accurately 85.7% of patient group (12 out 14), 83.3% of the control group (15 out 18) and 78.6% of relatives (11 out 14). This model remarkably improved the correct classification of the relatives compared with the previous models, presenting only 21.4% of incorrect classification (all of them in the control group).

#### *3.2.4. Prediction of group membership combining neuropsychological, social cognition and psychopathological data excluding PANSS scale*

Finally, we run a statistical analysis without PANSS scores in order to test the previous model excluding the possible psychotic symptoms' a priori segregation of controls vs. patients. Again, all the predictors were entered without a selection method. Only one discriminant functions was obtained with these predictors with a Wilks's $\lambda = 0.306$, $\chi^2$ (8) $= 49.12$; $p < 0.001$. This last model obtained 76.1% of correct global classification (67.4% cross-validated), grouping accurately the 78.6% of patient group (11 out 14), the 83.3% of the control group (15 out 18) and the 64.3% of relatives (9 out 14). Although PANSS scale contributes to the prediction in the previous analysis, the current model does not significantly differ to the previous one.

## 4. Discussion

### *4.1. Detection of underlying impairment in patients and relatives*

Our work replicates previous findings [5,13,14, 56] by demonstrating that schizophrenic patients performed worse than relatives and controls in most of the measures included here; however, it extends these findings showing that relatives presented impaired performance only in specific measures. Of interest, Faux pas and Premorbid Adjustment Scale performance suggest endophenotypic-like group differences with patients exhibiting the worst performance, controls showing highest scores and relatives performing in between.

The Faux pas impairment has been discussed extensively elsewhere ([49], also [54]). This deficit observed in patient and relatives is not likely to be explained neither by generalized intellectual functioning nor by speed processing, given that the three groups exhibited similar performance in the Raven test and TMT-A. Neither can be explained by comprehension difficulty of the stories comprised in the task, since the lack of difference for control-related stories indicates that all the subjects were equally able to discriminate between stories containing a Faux pas and stories with a minor conflict. Likewise, discrepancy scores evidenced similar performance when identifying that the speaker was not aware of the faux pas when a faux pas situation had occurred.

Instead, the faux pas impairment observed in patients and first-degree relatives might be due to an impaired processing of context-sensitive information. Detecting when a faux pas has occurred requires the integration of two representations: (1) that the person committing faux pas does not know that he or she should not say it (cognitive component); and (2) the empathic understanding of the listener's feelings (affective component). This coordination of multiple representations is a high level critical component for regulating behaviour in a context sensitive manner.

Nevertheless, this finding should take into account that it might be partially mediated by executive dysfunction evidenced by TMT-B impaired performance in the patient group. Likewise, it might be in some degree mediated by the working memory load of the task, although the memory demands of this test could have been diminished since subjects could refer to the text while responding.

In the Eyes Test, no differences were found for the intergroup evaluation. However, other studies have not found significant differences either [29,30]. This finding is consistent with other reports claiming that the Eyes Test may be subjected to less schizophrenia liability [57].

PAS performance was observed to be impaired not only in patients, but also in their healthy relatives. Both groups obtained lowers scored compared to controls subjects proving impaired social behaviour according to this scale. This finding is in agreement with Shapiro et al. [52] finding of poor global adjustment in schizophrenia patients and unaffected siblings across the life-span. In specific, probands global adjustment diminished after the early adolescence, while in controls and siblings the adjustment improved after late adolescence. Lower scores in our sample might be reflecting subtle alteration of the neurodevelopment, explaining odd social behaviour exhibited by patients, arising as a result of interaction of environmental and genetic traits shared by patients and their first degree relatives. Therefore, preservation of cognitive and social abilities is needed for a successful functioning in every neurodevelopmental stage. Furthermore, this is also in agreement with authors suggesting that social cognition deficit is related to psychosocial functioning [11] and symptomatology in the patient group [17].

Premorbid social functioning and Faux pas impairment in relatives suggest poor social functioning and impaired context-sensitive social cognition in probands and relatives. Schenkel et al. [51] suggested that high level components of social cognition impairment, in particular, linguistic context-sensitive social cognition, are associated to poor social functioning early in the life course of patients and relatives. The novel result described here is that both deficits can be found in healthy relatives suggesting that social cognition and behaviour might be considered a trait of the disease and could be related to vulnerability of the disease.

### 4.2. *Prediction of group based on cognitive, social cognition and psychopathological measures*

When psychiatric scales were analyzed, the best predictors were SIAPA gustative, PANSS Positive and Negative scale, and PAS score. These predictors performed accurately classifying subjects in each group, and were particularly helpful distinguishing patients from unaffected participants (both relatives and controls), which might be expected considering that these are measures with high predictive validity designed to detect altered behaviour in patients.

In the second model, where cognitive and social cognition were analyzed, the best predictors were IAT Face Compatible Accuracy and Faux Pas indicating that the ability to correctly categorize faces depicting happy and angry faces and the ability of detecting faux pas are the best predictors of group membership. Nevertheless, a higher percentage of misclassification emerged from the relative group in this model denoting social cognition similarities between patient and their unaffected relatives. The scatter plot of the discriminant space showed higher closeness of relatives and patient groups, suggesting that these types of task are robust markers of vulnerability of the disease.

When all the selected predictors were collapsed in one only model, the best predictors were a combination of two discriminant functions comprising the PANSS Positive and Negative, PAS, Faux Pas test, IAT face compatible accuracy. Even when PANSS was excluded form the analysis, similar effects were observed. This indicates that the best predictors of membership were the combinations of clinical symptoms, social functioning, context-sensitive social cognition and face categorizing. Although, the distance between patients and relatives centroids in the discriminant space was enlarged again, the most accurate classification percentage was achieved. Moreover, the relatives group received an improved classification in this model (78.6%, compared with the 42.9% of the previous comparison). This suggested that the combination of techniques, in the present study behavioural/psychiatric scales and neuropsychological/social cognition tasks, are the best approach for characterizing the disease. This finding is consistent with the advice from other laboratories recommending a composite approach for understanding the heterogeneous manifestations of this complex disease [50]. Our study has the limitation that does not permit to draw conclusions about differential contributions of genetic and environmental factors. Notwithstanding this, the data suggest that the high-level components of social cognition, as well as social behaviour should be considered as possible endophenotype candidates for future studies.

We chose psychopathology measures to assess also controls and unaffected relatives because recent reports have shown that these kinds of measures are sensitive for assessing vulnerability effects in first degree relatives. Psychopathological measures such as SIAPA, PAS or the Calgary scale have been effective for identifying the first degree relatives as a groups less affected than probands but more impaired than controls (e.g. [18,36,37,40]). Moreover, measures of psychotic symptoms such as the PANSS have been shown sensitive to discriminate relatives form both, controls and probands [10,26,36] and the same has been reported for other psychiatric disorders [32]. Those studies suggest that psychopathological measures exhibits familiar co-segregation, which is an important criterion for endophenotype agenda [7]. Consequently, we included psychopathological measures in order to assess vulnerability in the relative group. In addition we made two combining analysis wit and without the PANSS in order to reduce the a priori discriminative PANNS's effects (both analysis yield similar results). In brief, our data evidences a benefit by combining psychopathological, neuropsychological and social cognition measures to assess schizophrenia vulnerability.

### *4.3. Limitations*

This study can only be considered as preliminary since our sample size. Our results, obtained with a high number of tests in a small group of families with heterogeneous relatives must be replicated in a larger sample. Further studies assessing genetic polymorphisms would consider a reduced set of measures based on those witch discriminated more accurately between the three groups. Finally, future research is needed to examine whether symptomatology might explain a large proportion of social cognition variance in larger sample of healthy relatives.

## 5. Conclusion

Our results suggest that a specific combination of clinical, cognitive and social measures is the best approach to discriminate between controls, relatives and probands. This is an important topic since schizophrenia research is a clinically and cognitively heterogeneous condition. This heterogeneity can reduce the power and obscure the findings from studies to genome scans, clinical measures or neuropsychological studies. For instance, in endophenotype agenda, the use of combined and multilevel measures has been recommended (e.g. [27,31,41,61]). Moreover, in schizophrenia research there is a strong recommendation to include cognitive, clinical and social functions in further endophenotype and vulnerability studies [53,63]. Although preliminary, our data can be informative of which kind of multilevel measures should be included in further genetic studies with larger samples of participants.

## Acknowledgments

Funding for this study was provided by FINECO, CONICET and Diego Portales Grants; those institutions have no further role in study design; in the collection, analysis and interpretation of data; in the writing of the report; and in the decision to submit the paper for publication.

## Declaration of interest

None to declare.

## Supplementary data

### Measures

*Assessment of cognitive functioning*

*Raven's Coloured Progressive Matrices*
This multiple choice test measures intellectual capabilities [46]. The test has no time limit and comprises 36 items. Every item shows to the examinee a pattern with a part missing, the missed part is placed below the pattern amongst 5 distracters. The subject is asked to identify the missing part.

*Trail making test (TMT)*
TMT constitutes one of the most widespread measures of executive functioning. TMT part A requires to sequentially draw a line to connect 25 numbered circles in ascending order, and it measures speed processing. TMT part B asks to the subject to draw a line to connect 25 circles with letters and numbers, alternating between them. TMT-B measures sequencing and mental flexibility [6]. Both parts have to be completed as quickly as possible.

*Assessment of social cognition abilities*

*Reading the mind in the eyes*
Test designed by Baron- Cohen et al. [4] to assess the ability to recognize social emotions. It comprises 36 pictures of eyes depicting emotions surrounded by 4 alternatives of possible emotional states. The total scores is the number of emotions correctly identified.

*Faux pas test*
In this test, participants are read a story that may or may not contain a social faux pas [3,59]. The stories were placed in front of the participants, therefore reducing the demands on working memory. Ten stories contained a faux pas and the other ten stories did not contain a faux pas (Control stories). In the stimuli stories containing a faux pas, the character committing the faux pas was unaware that they had said something inappropriate, while the person in the story hearing it might have felt hurt or insulted. Performance was scored regarding the adequate identification of the faux pas and the adequate rejection of those stories which did not contain a faux pas. When a faux pas was correctly identified subjects were also asked two additional questions to measure intentionality – that is, recognizing that the person committing the faux pas was unaware that they had said something inappropriate– and emotional attribution, in which participants should recognize that the person hearing the faux pas might have felt hurt or insulted. Two additional memory questions checked that certain aspects of each story were retained.

*Emotional morphing* (*EM*)

Computational paradigm which measures dynamic recognition of six basic emotions: happiness, disgust, sadness, fear and anger [62]. In EM, the subject observes a face which progressively changes from a neutral face (0% expression) to intensely express an emotion (100% expression). The observer is asked to recognize the emotion as quickly and accurately as possible. The software Morpher 3.0 was used to create a sequence of 20 intermediate states of facial expression.

*Emotional implicit association task* (*Emotional IAT*)

IAT [20] is an experimental method that measures the strength of associations. The Emotional IAT is a modified version of the IAT, in this simultaneous stimulus categorization task the subject is asked to classify the emotional valence of positive/negative words and the emotional valence of positive/negative faces

The task involved the presentation of happy ($n = 20$) and angry ($n = 20$) faces along with pleasant words ($n = 71$) and unpleasant words ($n = 72$). Stimuli validation can be found in Hurtado et al. [25]. The test blocks comprised 100 stimuli. Faces were displayed for 100 ms and words for 300 ms. Some blocks had the same valence dimensions: negative (anger/unpleasant labels on one side of the screen) and positive (happiness/pleasant categories on the other). These were called compatible blocks. Blocks having the opposite association were called incompatible blocks. In this way, the incompatible blocks imply either an associative contextual effect of semantic interference with facial evaluation or an associative contextual effect of facial interference with semantic evaluation. The dependent variable were IAT score, which is a measure of interference effect, accuracy for categorizing compatible face/words, and accuracy for categorizing incompatible face/words.

*Psychiatric rating scales*

*Structured interview for assesing perceptual anomalies* (*SIAPA*)

SIAPA was developed by Bunney et al. [8] for evaluating senso- perceptual anomalies. SIAPA comprises 15 items, divided in 5 sub-scales looking up to auditive, visual, gustative, olfactive and tactile perceptual anomalies. Each sub-scale explores hipersensibility, inundation and selective attention to real stimuli. Every item is scored using a 5- points Likert scale assessing frequency of symptoms. These questions only include everyday and normal perceptual experience and it does not consider hallucinations, delusional experience or psychotic crisis.

*Positive and negative symptoms scale* (*PANSS*)

PANSS [29] is widely used instrument in the study of schizophrenia. It is a semi-structured interview which assess the presence the positive and negative symptoms, as well as general psychopathology. It comprises a 7-items scale for positive and negative symptoms, and a 16 items scale for general symptoms. Every item is scored using a 7-points scale according to the intensity of the symptom.

*Calgary depression scale for schizophrenia* (*CDSS*)

CDSS [1] is semi-structured interview designed to appraise depressive episodes in schizophrenia. It comprises 9 items: depression, hopelessness, self-depreciation, guilty ideas of reference, pathological guilt, morning depression, early morning waking, suicide, and observed depression. Each item is scored in a 4 points scale ranging from absent to severe. Final score is the sum of every item based on the observed behaviour during the interview.

*The premorbid adjustment scale* (*PAS*)

PAS [9] is a retrospective scale for assessing degree of achievement of developmental goals throughout infancy, adolescence and adulthood, if applicable. The scale is composed by 4 sub-scales for each stage assessing sociability, withdrawal, relationship with peers, and ability to establish intimate socio-sexual ties in each period. Besides, a general scale assesses factors like highest functioning reached by subject, level of energy, level of education, and independence. The performance is scored in a 7-points scale where 0 represents a healthy adjustment and 6 represents a pathologic development. Assessment period ends 6 months before first psychotic episode or first hospitalization

## Results

*Detection of cognitive, social cognition and clinical impairment in patients and relatives*

Mean scores of the three groups on neuropsychological tests, social cognition tasks and psychopathological scales are shown in Table 2. For neuropsychological tests, scores were analyzed by entering the scores into a One-way ANOVA comparing performance of patients, relatives, and controls. No group differences were found (F(2,45) = 1.51; $p = 0.32$), indicating similar intellectual functioning across the three groups.

In the TMT-A, no difference between the three groups were observed evidencing similar speed processing performance in this task (F(2,45) = 2.26; $p = 0.21$). However, in the TMT-B significant difference was found across the groups (F(2,45)4.33; $p < 0.05$). Tukey adjustments for multiple comparisons showed significant differences only between patients and controls.

For social cognition tasks, In the Faux Pas test, scores showed a significant difference between the three groups (F(2,45) = 9.26; $p < 0.001$). Tukey adjustments for multiple comparisons showed significant differences benefiting controls compared to patients ($p < 0.001$). Significant differences also existed between relatives and controls indicating a poorer performance of relatives ($p < 0.05$). No differences between patients and relatives were found ($p = 0.33$) indicating similar performance in both groups. In stories containing a minor conflict, but not a faux pas, no difference was observed across the groups evidencing adequate comprehension of the task in the three groups (F(2,45) = 0.56; $p = 0.61$).

For the Emotional IAT, significant differences were only evidenced by Accuracy Compatible Faces (F(2,45) = 3.54; $p < 0.05$) and Words (F(2,45) = 4.38; $p < 0.05$). Subsequent Tukey post hoc analysis showed significant differences between patients and controls (both, $p < 0.05$), evidencing an impaired performance in patients for categorizing faces and words with compatible emotional valence.

Dynamic recognition of emotions was examined using EM test, showing significant differences for Emotions (F(5,45) = 32.5; $p < 0.001$). However, no effect of groups, nor interaction effect were found (both, $p >$ 0.05)

For psychopathological measures, the performance in the PANSS was analyzed separately for the Positive, Negative and General sub-scales. The Positive subscale showed significant difference F(2,45) = 56.86; $p < 0.001$. Tukey post-hoc comparisons found difference between patient and controls ($p < 0.001$) and patient compared to relatives ($p < 0.001$). For Negative scale, a significant difference was observed (F(2,45) = 59.37; $p < 0.001$). Post hoc comparison showed larger impairment of patients compared to relatives and controls (both, $p < 0.001$). Regarding the General scale, significant difference was observed between the three groups (F(2,45) = 13.602; $p < 0.001$). Once again, post hoc analysis showed larger impairment of patients compared to relatives and controls (both, $p < 0.001$).

Senso-perceptual anomalies were analyzed entering the SIAPA scores in an ANOVA with Modality (visual, auditive, olfactive, tactile and gustative) as within-subject factor and Group as a between-subject factor (patient, relative and control). Only a main effect of group was found (F(2,45) = 7.97; $p < 0.01$) with patient exhibiting lower performance in comparison to control ($p > 0.01$) and relatives ($p < 0.05$).

## Supplementary tables

Supplementary Table 1

Standardized Coefficients of the canonical discriminant function for psychopathological predictors

| | Discriminant functions | |
|---|---|---|
| Predictor Variables | 1 | 2 |
| SIAPA – Gustative Scale | −0.475 | 0.457 |
| PANNS – Positive Scale | 0.774 | −0.357 |
| PANNS – Negative Scale | 0.638 | −0.790 |
| PAS | 0.128 | 1.216 |

Supplementary Table 2

Standardized coefficients of canonical discriminant functions for social cognition and cognitive predictors

| | Discriminant functions | |
|---|---|---|
| Predictor Variables | 1 | 2 |
| Faux Pas Score | 0.918 | −0.502 |
| IAT Conguent Face Accuracy | 0.752 | 0.729 |

Supplementary Table 3

Structure matrix for psychopathological, social cognition and cognitive predictors. Intra-group combined correlations between discriminant variables and typified canonical discriminant functions. Variables were ordered based on the size of the correlation with the function

| | Discriminant functions | |
|---|---|---|
| Predictor Variables | 1 | 2 |
| PANNS – Negative Scale | 0.808* | 0.005 |
| PANNS – Positive Scale | 0.790* | −0.021 |
| PAS | 0.633* | −0.556 |
| Faux Pas Score | −0.247 | 0.594* |
| IAT Conguent Face Accuracy | −0.174 | 0.282* |
| SIAPA – Gustative Scale | 0.177 | −0.190* |

*Highest absolute correlation between each variable and any discriminant function.
